# Non-Invasive, Targeted Nanoparticle-Mediated Drug Delivery across a Novel Human BBB Model

**DOI:** 10.3390/pharmaceutics15051382

**Published:** 2023-04-30

**Authors:** Shona Kaya, Bridgeen Callan, Susan Hawthorne

**Affiliations:** School of Pharmacy and Pharmaceutical Sciences, Ulster University, Coleraine BT52 1SA, N. Ireland, UK

**Keywords:** in vitro model, blood–brain barrier (BBB), nanoparticles, drug delivery, targeted receptor-mediated transcytosis, ligand conjugation, nicotinic acetylcholine receptor

## Abstract

The blood–brain barrier (BBB) is a highly sophisticated system with the ability to regulate compounds transporting through the barrier and reaching the central nervous system (CNS). The BBB protects the CNS from toxins and pathogens but can cause major issues when developing novel therapeutics to treat neurological disorders. PLGA nanoparticles have been developed to successfully encapsulate large hydrophilic compounds for drug delivery. Within this paper, we discuss the encapsulation of a model compound Fitc-dextran, a large molecular weight (70 kDa), hydrophilic compound, with over 60% encapsulation efficiency (EE) within a PLGA nanoparticle (NP). The NP surface was chemically modified with DAS peptide, a ligand that we designed which has an affinity for nicotinic receptors, specifically alpha 7 nicotinic receptors, found on the surface of brain endothelial cells. The attachment of DAS transports the NP across the BBB by receptor-mediated transcytosis (RMT). Assessment of the delivery efficacy of the DAS-conjugated Fitc-dextran-loaded PLGA NP was studied in vitro using our optimal triculture in vitro BBB model, which successfully replicates the in vivo BBB environment, producing high TEER (≥230 Ω/cm^2^) and high expression of ZO1 protein. Utilising our optimal BBB model, we successfully transported fourteen times the concentration of DAS-Fitc-dextran-PLGA NP compared to non-conjugated Fitc-dextran-PLGA NP. Our novel in vitro model is a viable method of high-throughput screening of potential therapeutic delivery systems to the CNS, such as our receptor-targeted DAS ligand-conjugated NP, whereby only lead therapeutic compounds will progress to in vivo studies.

## 1. Introduction

The central nervous system (CNS) is protected by a highly regulated physiological barrier known as the blood–brain barrier (BBB). The BBB consists of tightly regulated blood vessels formed by brain endothelial cells (BEC) and tight junction (TJ) proteins, forming part of the neurovascular unit (NVU), which also consists of pericytes, astrocytes, neurons and basement membrane (basal lamina) [1,2,3,4]. The endothelial cells that line the blood vessels of the BBB are unlike the endothelial cells that line blood vessels found within other organs of the body, as they are unfenestrated due to the presence of TJ proteins [2,3]. TJ proteins, such as occludin, claudin-5 and ZO-1, are expressed and regulated by cellular interaction and proximity between the endothelial cells and the other brain cell types that form part of the NVU and help prevent paracellular transport of toxins and pathogens. Even though TJ proteins are present within the BBB, the BBB facilitates the transportation of molecules that are essential for the maintenance of CNS homeostasis via diffusion (simple or facilitated), mediated or active transportation and the proximity of the circulating blood flow. However, due to the tightly regulated blood vessels of the BBB, paracellular diffusion is severely restricted and not a viable mode of transport for large hydrophilic molecules such as biologics (antibodies and proteins), causing issues with drug development, drug delivery and targeting of novel drugs for neurological conditions [1,2,4,5,6,7,8,9] Drugs that are lipid soluble and have a low molecular weight can transverse the BBB by transmembrane diffusion, which relies on the drug merging with the cell membrane by a non-saturable mechanism [1]. There can be issues however if the drug is highly lipid soluble, as some of the drug can remain in the cell, with only a small concentration of the drug reaching the CNS [1]. Transmembrane diffusion is not possible for therapeutics of high molecular weight and/or hydrophilic in nature; therefore, different mechanisms designed to transverse the BBB have been investigated. Currently, there are parenteral and non-parenteral routes of administration of drugs to treat neurological disorders, such as Parkinson’s disease (PD), Alzheimer’s disease (AD), dementia and stroke. Parenteral administrative routes for CNS delivery include intrathecal administration via intracerebroventricular (ICV) port, or intrathecal lumber (IT-L) injections or by convection-enhanced delivery (CED) such as polymeric implants [3,10]. Another method of administering a drug is peri-spinally using the cerebrospinal venous system (CSVS) [11]. Traditionally, these methods have a high success rate in terms of drug administration, but they are costly, requiring highly skilled clinicians and often hospitalisation for the patient as well as being uncomfortable and distressing for the patient. One such example of where invasive delivery prevents the full potential of a therapy is seen using etanercept.

Etanercept was first approved by the FDA in 1998 to treat rheumatoid arthritis. Etanercept is a potent anti-TNF (tumour necrosis factor) fusion protein and TNF inhibitor and was of interest for treating neuroinflammatory disorders such as stroke and Alzheimer’s disease. However, to treat the neuroinflammatory disorders within the CNS, etanercept (a protein with a molecular weight of 150 kDa that cannot be delivered systemically), must be administered peri-spinally and transverse the dura mater utilising the CSVS [12,13,14,15,16]. Reports by Tobinick et al. (2014) have shown that after one dose of peri-spinal etanercept, patients with long-term acute brain injuries had improved aphasia and apraxia and the left hemiparesis is reduced [14]. A study carried out by Ralph et al. (2020) showed rapid and significant results for chronic post-stroke management by administration of peri-spinal etanercept, which shows that peri-spinal administration of large molecules such as etanercept is effective [15]. Even though etanercept has the potential to successfully treat neurological inflammation, the invasive delivery of the treatment can cause issues for long-term suffers of AD or PD, such as severe discomfort and distress; therefore, non-invasive administration would be a better mode for BBB drug delivery and expand upon its current use [3].

Chemical modifications of drugs to aid in their administration via non-invasive routes are currently utilised to modify small molecular weight drugs to become more lipophilic and increase their ability to permeate the BBB [3]. L-3,4-dihydroxyphenylalanine (L-DOPA) is a non-invasive form of treatment for neurological disorders, specifically PD, and is taken orally in tablet form. Unlike dopamine, L-DOPA, which is a precursor of dopamine, transverses the BBB by system L (LAT1), which is a form of amino acid transporter, and once across the BBB it undergoes a decarboxylation reaction to the active form, dopamine. L-DOPA has been used to treat the motor symptoms caused by PD for over 50 years [17,18,19,20,21].

One such mechanism that could be used to increase patient compliance, and reduce off-site targeting, toxicity and side effects, is the non-invasive method of drug delivery by an active transport mechanism known as receptor-mediated transcytosis (RMT). RMT transports molecules across the BBB into the CNS by targeting the receptors on the BEC; this is normally achieved either by preparing a complex between the drug of interest and the receptor-targeting entity, or by encapsulating the drug within a nanocarrier with the RMT-targeting ligand on the surface of the carrier [7,22,23,24]. Nanocarriers such as nanoparticles (NP) can be chemically modified by the conjugation of ligands to the surface of the loaded NP, which can then target the receptors on the BEC and facilitate the transportation of the novel drug across the BBB by RMT [25,26]. NP have the added benefit of protecting their internalised cargo, such as proteins and antibodies, from degradation by endogenous compounds. In addition, encapsulating compounds within the NP increases the accumulation of the therapeutic drug at the target site, increasing efficacy and requiring a reduction in drug dose, thereby reducing off-site targeting effects [22,23,24,27,28,29]. 

Ligand-conjugated NP have ligands attached which target receptors that are known to be found on the surface of BEC [26]; prior to any in vivo assessment, analysis of a variation in ligand-conjugated NP with different drug load concentrations and/or different ligand types or amounts needs to be analysed. Therefore, there is a requirement for the most cost-effective, time-efficient and ethical way to carry out this preliminary assessment by using in vitro BBB models where possible.

When developing new therapeutics, in vitro BBB models have been widely utilised to investigate drug targeting, drug permeability and drug toxicity. In vitro models can be constructed in various ways depending on cost, cell source (immortalised, primary or stem cells) and time frame, to mimic the in vivo physiology and architecture of the BBB. The in vitro models can be developed to maintain high integrity and low permeability by paracellular transport. There are different forms of in vitro models such as organoids/spheroids which are mainly developed using stem cells, microfluidic devices or transwell models which can be developed using primary, stem or immortalised cells [30,31,32,33]. However, both organoid/spheroids and micro-fluidic models are time-consuming, costly and complex, with microfluidic devices having limited scalability and prone to errors, whilst organoid/spheroid models have poor BEC coverage and poor vascularisation [30,31,32,33]. Transwell models are generally cost-effective, scalable, adaptable and easily produced, and have shown to be effective for high-throughput drug screening and are therefore a viable model to utilise when assessing the efficacy of novel compounds prior to lead compound in vivo analysis [34,35,36]. The inclusion of shear stress, cellular substrata and the co-localisation of astrocytes and pericytes produces an in vitro model which more accurately mimics the in vivo BBB architecture by enhancing TJ protein expression, increasing barrier integrity and thereby decreasing non-specific permeability. When developing drugs for the treatment of neurological disorders, the use of in vitro models such as transwell models is an important tool to enhance the development of targeted drug delivery systems (DDS) that have the ability to traverse the BBB via RMT, ensuring that novel neurological therapeutics can be delivered to the required site as efficiently and non-invasively as possible.

In this article, we demonstrate the development and optimisation of a novel, in vitro human-immortalised cell BBB model and also demonstrate that receptor-targeting PLGA NP are an ideal vehicle for transporting large, hydrophilic molecules across the BBB in vitro. We also demonstrate that the chemical modification of the surface of NP by a targeting ligand, aids in the transport of the loaded NP across the BBB via RMT. We have shown this by encapsulating Fitc-dextran, a large hydrophilic molecule (70,000 Da), within a PLGA NP and conjugating DAS peptide to the surface of the NP. DAS (NH_2_-GGGGSGCLRVGGRrRrRr-COOH) is a ligand that we previously designed [37] which aids in the transportation of the loaded NP across the BBB by RMT, due to the DAS affinity for alpha 7 nicotinic acetylcholine receptors (α-7 nAChR), which are found on the membranes of the BEC of the BBB. 

## 2. Materials and Methods

### 2.1. Cell Culture

#### 2.1.1. Materials

##### Cell Culture

Immortalised human brain microvascular endothelial cells (I-HBMEC) (Innoprot, Bizkaia, Spain) (P10361-1M), Immortalised human astrocytes (IA) (Innoprot, Bizkaia, Spain) (P10251-1M), CLTH/Immortalised Pericytes (IP) (Amsbio, Oxfordshire, U.K.) (CL05008-CLTH), D-MEM/F:12 (1:1) (CE) (Thermofisher, Cambridge, U.K.) (11320074), Insulin-trans-sel-G, 100× (Thermofisher, Cambridge, U.K.) (41400045), Penicillin streptomycin (PS) (Gibco, Paisley, U.K.) (15070-063), Fetal bovine serum (FBS) (Gibco, Paisley, U.K.) (A3160801), Hydrocortisone solution (50 um) (Merck, Dorset, U.K.) (H6909), Fibroblast growth factor-basic (BFGF) (Merck, Dorset, U.K.) (SRP3043), Heparin sodium salt (Merck, Dorset, U.K.) (H3149), Dulbecco’s phosphate buffered saline, M (DPBS) (Merck, Dorset, U.K.) (D8537), Accutase^®^ solution (Merck, Dorset, U.K.) (A6964), Tissue culture flask, 75 cm^2^ growth area (T75) (Sarstedt, Leicestershire, U.K.) (83.3911.002).

##### Transwell Model

Corning TW PC membrane 6.5 mm, 0.4 µm, TCT, S (Merck, Dorset, U.K.) (CLS3413-48EA), Tissue culture, 24-flat-well sterile plate (Sarstedt, Leicestershire, U.K.) (83.3922), Magnesium sulphate (MgSO_4_) (Merck, Dorset, U.K.) (63136), Fibronectin bovine plasma (Merck, Dorset, U.K.) (F1141), Gelatin solution Bioreagent, Type B, 2% (Merck, Dorset, U.K.) (G1393). 

#### 2.1.2. Methods

##### Preparation of Cells for In Vitro EP + A transwell BBB Model

I-HBMEC, IP and IA were all seeded and grown in T75 flask with DMEM/F:12 medium supplemented with 10% FBS, 1% PS, 5 mL hydrocortisone solution, 2.5 mL Insulin-trans-sel-G, 100×, 30 µL BFGF, 15 mg of 100 KU Heparin sodium salt. Cells were aspirated and washed with DPBS, detached with Accutase and centrifuged using a Thermo Scientific Medifuge centrifuge, at 1000 rpm (I-HBMEC & IA) or 900 rpm (IP) for 5 min for formation of cell pellet.

##### Preparation of In Vitro Tri-Culture Transwell BBB Model

Day-2: The underside of the transwell polycarbonate inserts was precoated with 40 µL substratum (30 µg/mL fibronectin solution and 10 µg/mL gelatin solution). The precoated inserts were incubated for 4 h at 37 °C 5% CO_2_. The underside of inserts was washed thrice with DPBS and inserts were inserted into wells on a 24-well plate that contained 200 µL DPBS to prevent underside of insert drying out. In apical layer of insert, 100 µL of substrata solution was pipetted and then incubated overnight at 37 °C 5% CO_2_.

Day-1: Inserts and wells on 24-well plate were washed thrice with DPBS and allowed to airdry at RT. The IP and IA pellets were diluted to 8 × 10^4^ cells/mL in complete medium containing 10 mM MgSO_4_ (Mg^2+^). Equal volumes of each 8 × 10^4^ cells/mL IA and IP were combined to form 4 × 10^4^ cells/mL IA/IP cell solution. Transwell inserts were inverted and 40 µL of the IA/IP cell solution was pipetted onto the pre-coated underside of inserts and incubated at RT for 40 min to allow cells to adhere. Then, 600 µL of the medium, was pipetted into wells on well plate and inserts were placed right side up into these wells, and 100 µL of the medium was pipetted into the apical layer of inserts and the plate was incubated overnight at 37 °C 5% CO_2_.

Day 0: The I-HBMEC pellet was diluted to 6.25 × 10^4^ cells/mL with medium. Apical layer of inserts was aspirated and 200 µL of the 6.25 × 10^4^ cells/mL of I-HBMEC was pipetted into the apical layer of insert, and the medium in basolateral layer (well of well plate) was aspirated and replaced with fresh medium containing Mg^2^+. Plate was incubated for 48 h at 37 °C 5% CO_2_ with gentle shaking at 100 rpm on a Grant-bio Orbital shaker PSU-10i.

Day 1: Allowed cells to adhere to insert surfaces.

Days 2–5: Measured trans-endothelial electrical resistance (TEER) daily using WPI EVOM 2 voltohmmeter with WPI electrode set for EVOM and replaced medium in basolateral and apical layer of model with fresh medium then incubated at 37 °C 5% CO_2_ at 100 rpm.

**Figure 1:**
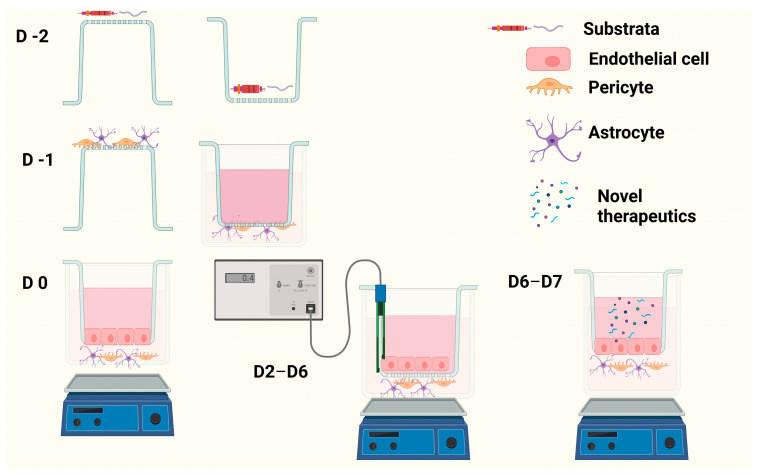
Image displays how the BBB model is developed over a 9-day period, including the application of shear stress by orbital shaker and TEER measurement using chopstick-style electrodes and a voltohmmeter (image created in BioRender).

Day 6: Measured TEER, then tested compounds of interest on BBB model, replacing medium with serum-free medium (SFM) containing test compound in apical layer. In basolateral layer, the medium was replaced with 600 µL SFM. Plate was incubated at 37 °C 5% CO_2_ at 100 rpm and samples were taken every half hour for 7 h and then at 24 h from basolateral layer. The medium removed from basolateral layer was replaced with an equivalent volume of SFM to maintain sink conditions (Figure 1).

### 2.2. Evaluation of Barrier Integrity

#### 2.2.1. Materials

MES hydrate (Merck, Dorset, U.K.) (M8250), N-(3-Dimethylaminopropyl)-N′-ethylcarbodiimide hydrochloride (EDC)(Merck, Dorset, U.K.) (E7750), N-Hydroxysuccinimide (NHS) (Merck, Dorset, U.K.) (130672), Fitc-CM-Dextran (Merck, Dorset, U.K.) (74817), DAS (GL Biochem Ltd., Shanghai, China), Dialysis tubing, benzoylated (Merck, Dorset, U.K.) (D7884).

#### 2.2.2. Methods: Preparation of DAS-Labelled Fitc-Dextran

A 25 mM MES buffer was prepared, and the pH was adjusted to pH 5–6. 30 mg/mL EDC, and 60 mg/mL NHS was added to the 5 mL 25 mM MES buffer and vortexed. Then, 10 mg/mL Fitc-CM-dextran was resuspended in 25 mM MES buffer and vortexed to aid dispersal. Then, 1 mL of EDC/NHS in 25 mM MES buffer was added to 1 mL of the 10 mg/mL Fitc-CM-dextran in 25 mM MES buffer, covered in foil and shaken at room temperature (RT) for 1 h to activate free carboxyl groups, and then 1.4 mg of DAS was added to 500 µL of ddH_2_O and vortexed to aid dissolution. The DAS solution was then added to the activated Fitc-dextran solution and incubated for 8 h at RT whilst shaking, to allow conjugation of DAS. This solution was dialysed overnight at RT, whilst shaking. This was then freeze-dried (Labconco, Freezone 4.5 Plus) for 48 h and stored at −20 °C until needed.

#### 2.2.3. Measurement of Barrier Permeability

On day 6 of BBB model, 1 mg/mL of Fitc-CM-dextran and 1 mg/mL of DAS-Fitc-dextran were resuspended in SFM, and 200 µL of each compound in SFM was added to the apical side of BBB model and incubated at 37 °C 5% CO_2_ at 100 rpm. Then, 150 µL of samples was removed from the basolateral side of each in vitro model every 30 min for 7 h and at the 24 h timepoints, and the fluorescence of each sample was analysed utilising BMG LABTECH FLUOstar Omega platereader (exc. 485 nm, em. 520 nm). Amount removed from basolateral side of in vitro model was replaced with SFM to maintain sink conditions, maintaining the basolateral volume at 600 µL. Percentage of compound to permeate the BBB was calculated using Equation (1).

(1) $$\%\mathrm{EE}=\frac{\mathrm{Mass}\;\mathrm{of}\;\mathrm{drug}\;\mathrm{added}-\mathrm{Mass}\;\mathrm{of}\;\mathrm{drug}\;\mathrm{in}\;\mathrm{supernatant}}{\mathrm{Mass}\;\mathrm{of}\;\mathrm{drug}\;\mathrm{added}}\times 100$$

### 2.3. Immunocytochemistry

#### 2.3.1. Materials

Anti-GFAP Alex fluor^®^488 (Invitrogen, Cambridge, U.K.) (53-9892-82), rabbit anti-α SMA (Abcam, Cambridge, U.K.) (ab5694), goat anti-rabbit TRITC (Abcam, Cambridge, U.K.) (ab6718), Poly-L-Lysine (Merck, Dorset, U.K.) (P4832), DPBS (Merck, Dorset, U.K.) (D8537), Methanol (Merck, Dorset, U.K.) (34860), Bovine serum albumin (BSA) (Merck, Dorset, U.K.) (A2153), DAPI readymade solution (Merck, Dorset, U.K.) (MBD0015), Slow fade™ Diamond antifade mounting medium (Invitrogen, Cambridge, U.K.) (S36967).

#### 2.3.2. Methods

Briefly, 13 mm round glass coverslips pre-coated with poly-L-lysine were incubated overnight at 37 °C 5% CO_2_. They were then washed with DPBS and seeded with 200 µL of 2 × 10^5^ cells/mL of IA and IP and incubated for 48 h at 37 °C 5% CO_2_. Media was aspirated and coverslips washed thrice with DPBS. Cells were fixated for 5 min with 100% methanol (−20 °C) and washed thrice with ice-cold DPBS. Coverslips were incubated cell faced down onto 100 µL drops of 1% BSA in DPBS for 1 h at RT. Coverslips were washed thrice with DPBS and incubated face down onto 50 µL drops of 1:5 dilution of rabbit anti-α SMA antibody in 1% BSA for 1.5 h in a humidified chamber. Coverslips were washed thrice with DPBS and incubated face down onto 1:10 dilution of anti-GFAP Alex fluor^®^488 antibody and 1:100 dilution of goat anti-rabbit TRITC antibody all in 1% BSA for 1.5 h in a dark humidified chamber, total volume of each drop being 50 µL. Coverslips were washed thrice with DPBS and incubated face down onto 50 µL drops of 300 nM DAPI in ddH_2_O solution for 2 min, in a dark chamber. Coverslips were washed thrice with DPBS and mounted onto slides using Slow fade™ Diamond antifade mounting medium, sealed and stored in dark at 4 °C until analysis. Analyses of the samples were completed by using Nikon Eclipse E400 microscope.

### 2.4. Protein Expression

#### 2.4.1. Materials

Trizma^®^ hydrochloride (Tris) (Merck, Dorset, U.K.) (10812846001), Sodium chloride (NaCl) (Merck, Dorset, U.K.) (71383), DPBS (Merck, Dorset, U.K.) (D8537), Sodium deoxycholate (Merck, Dorset, U.K.) (D6750), Triton 100 (Merck, Dorset, U.K.) (×100), Protease inhibitor cocktail (Merck, Dorset, U.K.) (P8340).

#### 2.4.2. Methods

Protein lysis buffer was prepared using 10 mM tris, 150 mM NaCl, 0.5% sodium deoxycholate and 0.5% triton 100 to 20 mL ddH_2_O and stored at 4 °C. Media were aspirated from the cells and then rinsed with DPBS. Then, 500 µL of DPBS was added to cells, and surface was scraped using a cell scraper and sample was pipetted into a microcentrifuge tube. The sample was centrifuged for 3 min at 800 rpm using Hettich Zentrifugen MIKRO 120. Supernatant was aspirated and the pellet was resuspended in 250 µL of protein lysis buffer. The sample was placed on ice for 20 min and then centrifuged for 5 min at 3000 rpm. Supernatant was removed and pipetted into a fresh microcentrifuge tube and 25 µL protease inhibitor cocktail was added, and supernatant was stored at −20 °C until analysis.

#### 2.4.3. SDS-PAGE and Western Blotting

##### Materials

Trizma^®^ base (Merck, Dorset, U.K.) (93350), Glycerol (Merck, Dorset, U.K.) (G5516), Sodium dodecyl-sulfate (SDS) (Merck, Dorset, U.K.) (11667289001), β-mercaptoethanol (Merck, Dorset, U.K.) (444203), Bromophenol blue (Merck, Dorset, U.K.) (114391), NUPAGE MOPS SDS running buffer (20×) (Thermofisher, Cambridge, U.K.) (NP0001), NUPAGE transfer buffer (20×) (Thermofisher, Cambridge, U.K.) (NP00061), NUPAGE nitrocellulose membrane filter paper sandwich (Thermofisher, Cambridge, U.K.) (LC2001), NUPAGE 10% Bis-Tris gel (1.0 MM 10 w) (Thermofisher, Cambridge, U.K.) (NP0301BOX), See Blue^®^ Plus 2 Prestained standard (Invitrogen, Cambridge, U.K.) (LC5925), Bovine serum albumin (BSA) ( Merck, Dorset, U.K.) (A2153), ZO1 antibody (Abcam, Cambridge, U.K.) (ab276131), Actin antibody (Santa Cruz, Heidelberg, Germany) (40549), Anti-rabbit IgG (whole molecules)-Alkaline phosphatase antibody produced in goat (Merck, Dorset, U.K.) (A3687), BCIP/NBT solution (Merck, Dorset, U.K.) (B6404). 

##### Methods

Reducing sample treatment buffer (×5) (RSTB) was prepared by combining 1.25 mL of stacking gel buffer (499 mM Trizma^®^ base pH 6.8), 385 mM SDS, 4 mL glycerol, 2.5 mL β-mercaptoethanol, 1.25 mL ddH_2_O and a few grains of bromophenol blue and stored at −20 °C. Then, 20 µL of cell lysate samples was pipetted into a microcentrifuge tube and a 1:5 dilution of RSTB was added to each sample. The samples were then heated to 100 °C for 10 min and then electrophoresed on a NUPAGE 10% Bis-Tris gel (1.0 MM 10 w) using ×1 NUPAGE MOPS SDS running buffer. The proteins were transferred onto NUPAGE nitrocellulose membrane using ×1 NUPAGE transfer buffer. 

The nitrocellulose membrane was blocked using 5% BSA in TBS for 2 h at RT. The nitrocellulose membrane was washed twice for 5 min in TBS, then 1:1000 dilution of anti-ZO1 antibody in 5% BSA in TBS and a 1:1000 dilution of anti-actin antibody in 5% BSA in TBS was added to the nitrocellulose membrane and incubated overnight, shaking at 4 °C. The nitrocellulose membrane was washed for 5 min twice in TBS and the incubated at RT for 2 h in 1:20,000 dilution of anti-rabbit IgG (whole molecules)-alkaline phosphatase antibody in 5% BSA in TBS solution and shaken. The wash step was repeated and BCIP/NBT substrate solution was added to the nitrocellulose membrane until purple bands appeared. Blot was scanned and analysed using Image J programme.

### 2.5. Nanoparticles (NP)

#### 2.5.1. Nanoparticle Formulation

##### Materials

Resomer^®^ Rg 502H Poly(D,L-lactide-co-glycolide) (PLGA) (Merck, Dorset, U.K.) (719897), Poly(vinyl alcohol) (PVA) (Merck, Dorset, U.K.) (363081), Dichloromethane (DCM) (Merck, Dorset, U.K.) (66742) and Fluorescein isothiocyanate-dextran (Fitc-Dextran) (Merck, Dorset, U.K.) (46945).

##### Methods

Briefly, 4 mg of Fitc-Dextran was added to 500 µL ddH_2_O and added dropwise to PLGA solution (100 mg of PLGA dissolved in 4 mL DCM). The Fitc-dextran/PLGA solution was then sonicated for 60 s at 80% amplitude using Fisher scientific ultrasonic homogeniser CL-18 to form a w/o emulsion. The w/o emulsion was added dropwise to a 1.25% PVA solution (15 mL) and sonicated for 2 min at 80% amplitude to form a w/o/w emulsion. The w/o/w emulsion was placed onto a magnetic stirrer overnight in the dark at RT to allow DCM evaporation. The emulsion was centrifuged at 18,809× *g* at 4 °C for 30 min using Sigma^®^ centrifuge 3–30 K. The supernatant was aspirated and stored at −20 °C for encapsulation efficiency analysis. The pellet was washed thrice with ddH_2_O and resuspended in 5 mL ddH_2_O, and placed in −80 °C for 2 h. Once frozen, the NP were placed into Labconco Freezone 4.5 Plus freezedryer, for 48 h. NP were stored at −20 °C until sample sizing and PDI analysis.

#### 2.5.2. Sizing and PDI

##### Materials

0.4 µM Minisart^®^ syringe filter (Sartorius, Surrey, U.K.) (16555), Malvern disposable folded capillary cells DT51070 (Malvern, Worcestershire, U.K.).

##### Methods

Briefly, 1 mg of DAS-FD-NP or FD-NP were resuspended in 1 mL ddH_2_O to make a 1 mg/mL solution. The 1 mg/mL solution was vortexed and pipetted into folded capillary cells and analysed on the Malvern zetasizer Nano series at 10 °C with 5 series (15 runs each series) to obtain size and PDI of the NP.

#### 2.5.3. Encapsulation Efficiency

##### Materials

Fluorescein isothiocyanate-dextran (Fitc-dextran) (Merck, Dorset, U.K.) (46945).

##### Methods

A calibration curve was produced using Fitc-dextran in ddH_2_O (exc. 485 nm, em. 520 nm, gain 750). The linear equation gained from the calibration curve was used to analyse the concentration of Fitc-Dextran within retained supernatant (Section 2.5.1. The encapsulation efficiency (EE) of Fitc-Dextran within the FD-NP was then determined by Equation (2).

(2) $$\%\mathrm{EE}=\frac{\mathrm{Mass}\;\mathrm{of}\;\mathrm{drug}\;\mathrm{added}-\mathrm{Mass}\;\mathrm{of}\;\mathrm{drug}\;\mathrm{in}\;\mathrm{supernatant}}{\mathrm{Mass}\;\mathrm{of}\;\mathrm{drug}\;\mathrm{added}}\times 100$$

#### 2.5.4. Release Assay

##### Materials

3,3-dimethylglutaric acid (Merck, Dorset, U.K.) (D4379), Sodium hydroxide (NaOH) (Merck, Dorset, U.K.) (221465), Sodium chloride (NaCl) (Merck, Dorset, U.K.) (71383), DPBS (Merck, Dorset, U.K.) (D8537).

##### Methods

Briefly, 0.01 M DMGA buffer was prepared using 6 mM 3,3-dimethylglutaric acid, 3.9 mM NaOH, 150 mM NaCl_2_ in 100 mL ddH_2_O, pH 4.5. 1.5 mg of FD- NP was resuspended in either 1 mL DMGA buffer or 1 mL DPBS and incubated at 37 °C. At each hourly time point (for 7 h then 24 h), the samples were centrifuged at 5000 rpm for 5 min using Hettich Zentrifugen MIKRO 120. Then, 150 µL of each sample’s supernatant was removed and replaced with 150 µL of fresh buffer and pellet redispersed in solution and incubated at 37 °C. The fluorescence of each sample was analysed (exc. 485 nm, em. 520 nm, 700 gain). The concentration of Fitc-Dextran released from NP was calculated using linear equation obtained from calibration curves and cumulative release results. 

#### 2.5.5. Conjugation Efficiency

##### Materials

DAS (GL Biochem Ltd., Shanghai, China), Phosphate buffered saline (PBS) (Dulbecco A) (Thermofisher, Cambridge, U.K.) (BR0014G), nd Pierce™ BCA protein assay kit (Thermofisher, Cambridge, U.K.) (23227).

##### Methods

DAS was conjugated to the surface of the FD-NP following the protocol published by Huey et al. (2019) [37].

#### 2.5.6. Delivery across BBB Model

##### Materials

Mecamylamine (Merck, Dorset, U.K.) (M9020), hexamethonium chloride (Merck, Dorset, U.K.) (H2138), anti-Nicotinic acetylcholine receptor antibody alpha 7/CHRNA7 antibody (α-7 nAChR antibody) (Santa Cruz, Heidelberg, Germany) (sc-58607).

##### Methods

The in vitro BBB model was prepared as shown in Section 2.1.2. On day 6 of the in vitro BBB model, 100 µL of either 2 mM mecamylamine in SFM, 2 mM hexamethonium in SFM, or 1:100 dilution of α-7 nAChR antibody in SFM was added to the apical side of transwell inserts and incubated for 1 h at 37 °C 5% CO_2_. Then, 0.4 mg/mL of Fitc-CM-dextran and 0.4 mg/mL of DAS-FD-NP were resuspended individually in SFM and 200 µL was added to the apical side of transwell insert containing antibodies or antagonist (which diluted NP concentration to 0.2 mg/mL). The models were incubated at 37 °C 5% CO_2_ at 100 rpm until each hourly time point. Then, 150 µL samples were removed from the basolateral side of each in vitro model for 7 h and at the 24 h timepoints and the fluorescence of each sample was analysed (exc. 485 nm, em. 520 nm). The media removed from basolateral layer of the model were replaced with SFM to maintain sink conditions, maintaining the basolateral volume at 600 µL. Percentage of drug to permeate BBB was calculated using Equation (1) (Section 2.2.3).

## 3. Results

### 3.1. Determining Transwell Model Architecture

**Figure 2:**
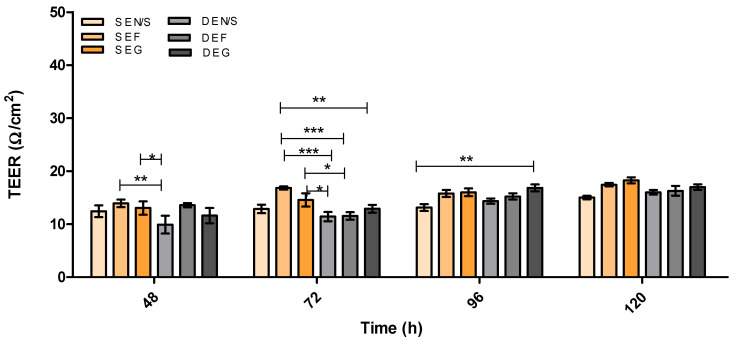
TEER (Ω/cm^2^) of static (S) versus dynamic (D) HMEC monolayer in vitro models utilising different substratum over 120 h. E = Endothelial cells, N/S = No substratum, F = Fibronectin and G = Gelatin. *n* = 9 ± SD, * = *p* < 0.05, ** = *p* < 0.01, *** = *p* < 0.001; statistical analysis was performed using two-way ANOVA Bonferroni post hoc tests.

Figure 2 demonstrates the effect of substratum and shear stress on the integrity of a monolayer of I-HBMECs (cell concentration of 6.25 × 10^4^ cells/mL) using TEER measurement. The I-HBMEC concentration was determined from previous proliferation studies.

**Figure 3:**
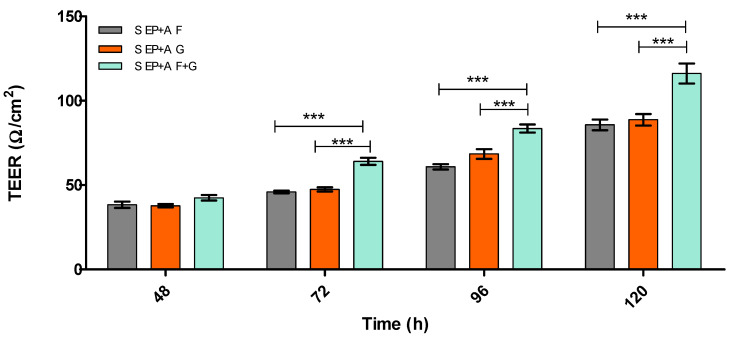
TEER (Ω/cm^2^) of static (S) EP + A in vitro BBB model over 120 h on different substrata. Endothelial cells (E), Pericytes (P) and Astrocytes (A), Fibronectin (F), Gelatin (G) Fibronectin + Collagen (F + C), Fibronectin + Gelatin (F + G), *n* = 9 ± SD, *** = *p* < 0.001; statistical analysis was performed using two-way ANOVA Bonferroni post hoc tests.

**Figure 4:**
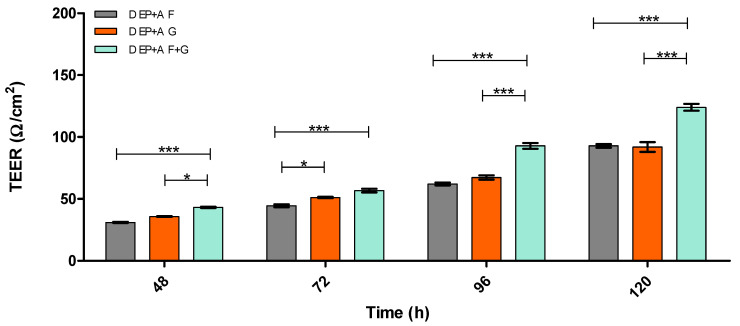
TEER (Ω/cm^2^) of dynamic (D) EP + A in vitro BBB model over 120 h on different substrata. Endothelial cells (E), Pericytes (P) and Astrocytes (A), Fibronectin (F), Gelatin (G) Fibronectin + Collagen (F + C), Fibronectin + Gelatin (F + G), *n* = 9 ± SD, * = *p* < 0.05, *** = *p* < 0.001; statistical analysis was performed using two-way ANOVA Bonferroni post hoc tests.

Figure 3 and Figure 4 demonstrate the integrity of the BBB model using the two optimal substrata from Figure 2, on their own or in combination, in both static and dynamic modes, using TEER measurement.

**Figure 5:**
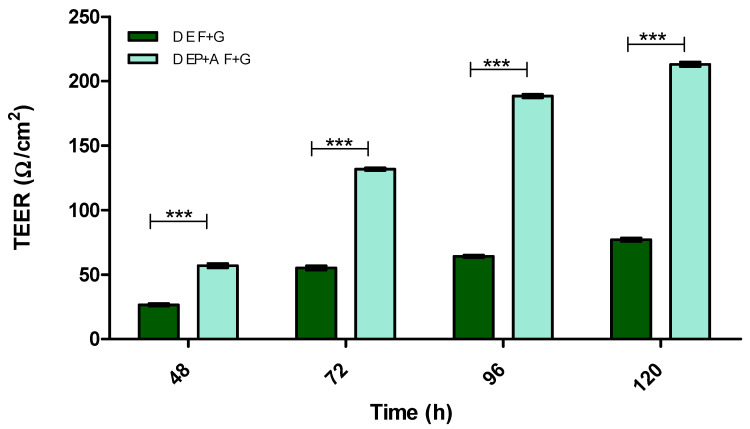
TEER (Ω/cm^2^) of dynamic (D) mono- and tri-culture in vitro BBB models over 120 h on Fibronectin + Gelatin (F + G) substratum combination, Endothelial cells (E), Pericytes (P) and Astrocytes (A), *n* = 9 ± SD, *** = *p* < 0.001; statistical analysis was performed using two-way ANOVA Bonferroni post hoc tests.

Figure 5 demonstrates how increasing cell concentration of IA and IP from 2 × 10^4^ cells/mL to 4 × 10^4^ cells/mL influences BBB integrity (TEER). It also demonstrates how adjusting substrata placement can influence BBB integrity of the monoculture model.

**Figure 6:**
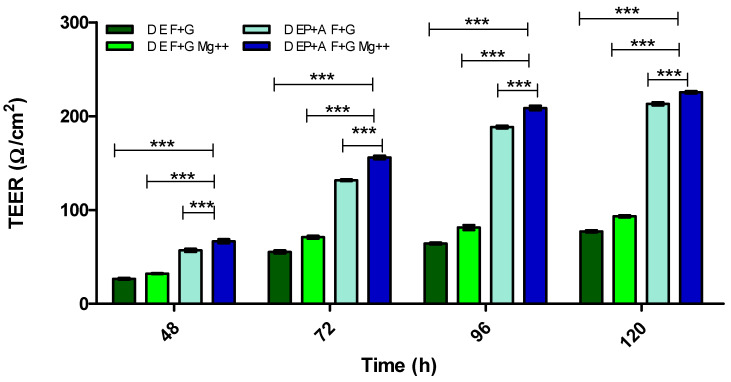
TEER (Ω/cm^2^) of two different dynamic (D) in vitro BBB models over 120 h with and without magnesium (Mg^2+^). Fibronectin + Gelatin (F + G), Endothelial cells (E), Pericytes (P) and Astrocytes (A). *n* = 9 ± SD, *** = *p* < 0.001; statistical analysis was performed using two-way ANOVA Bonferroni post hoc tests.

Figure 6 demonstrates the effect Mg^2+^ has on BBB integrity of a mono- and triculture model using TEER measurement.

**Figure 7:**
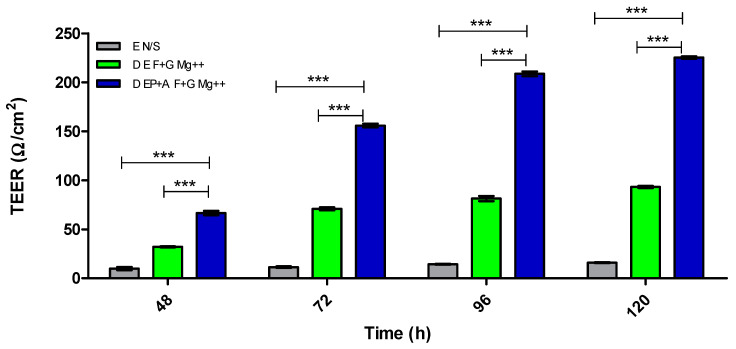
TEER (Ω/cm^2^) of dynamic (D) models: endothelial monolayer N/S, endothelial monolayer F + G Mg^2+^ and EP + A F + G Mg^2+^. N/S = no substratum, E = endothelial cells, P = pericyte, A = astrocyte, F + G = fibronectin + gelatin and Mg = magnesium. *n* = 9 ± SD, *** = *p* < 0.001; statistical analysis was performed using two-way ANOVA Bonferroni post hoc tests.

Figure 7 demonstrates the distinct differences between a monolayer with no substratum, and a monolayer with combined substratum F + G and Mg^2+^ to that of the optimal in vitro BBB model (D EP + A F + G Mg^2+^) using TEER measurement. 

### 3.2. Evaluation of Barrier Integrity

**Figure 8:**
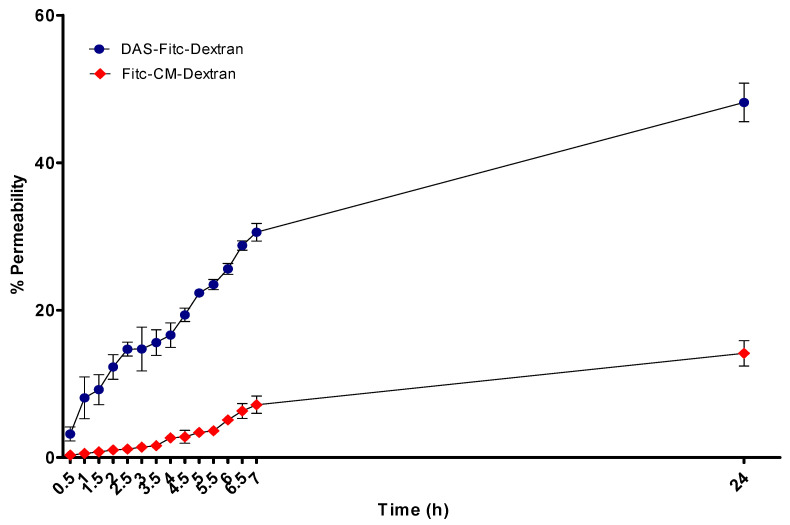
The percentage permeability of DAS-Fitc-dextran and Fitc-dextran on our in vitro BBB model, over 24 h (exc. 485 nm, em. 520 nm), *n* = 9 ± SD.

Figure 8 demonstrates the integrity of the optimal in vitro BBB model (D EP + A F + G Mg^2+^) over 24 h. This was determined by measuring the percentage (%) of Fitc-CM-dextran versus DAS-Fitc-dextran, to traverse the in vitro BBB by fluorimetry (exc. 485 nm, em. 520 nm).

**Figure 9:**
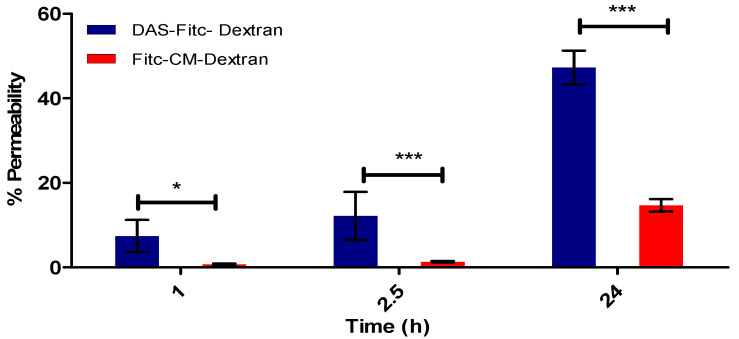
Graph demonstrating, permeability at specific time points between DAS-Fitc-dextran and Fitc-dextran using optimal in vitro BBB model. *n* = 9, 2-way ANOVA, * significant difference of *p* < 0.05, *** significant difference of *p* < 0.001 between permeability of fluorescent compounds at same time point.

Figure 9 demonstrates the significant difference in permeability of Fitc-CM-dextran and DAS-Fitc-dextran, in the in vitro BBB at specific time points of (1 h, 2.5 h and 24 h). Statistical analysis was performed by two-way ANOVA.

### 3.3. Immunocytochemistry

**Figure 10:**
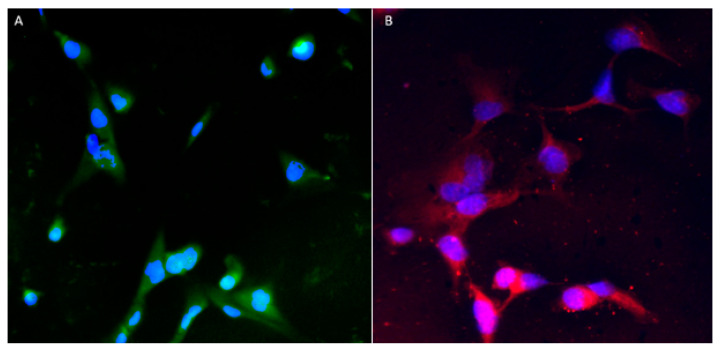
Immunocytochemistry imaging of (**A**) immunofluorescence of GFAP proteins expressed on IA (green signal) and (**B**) immunofluorescence of α SMA proteins expressed on IP (red signal). Both cells’ nuclei were counter-stained with DAPI (blue signal).

Figure 10 shows IA (Figure 10A) and IP (Figure 10B) growing together, demonstrating the ability of the cells to co-localise (a necessity for our EP + A BBB model). Anti-α SMA primary antibody was used to detect IP (which are known to produce α SMA proteins) and a secondary goat anti-rabbit TRITC (exc. 547 nm, em. 572 nm) antibody conjugate, which produces a red signal that could be detected under a fluorescence microscope. An anti-GFAP antibody Alex fluor^®^488 (exc. 499 nm, em. 520 nm) conjugate was used to detect glial fibrillary acidic protein (GFAP) which is expressed exclusively by astrocytes to produce a green signal under a fluorescence microscope.

### 3.4. Protein Expression

#### SDS-PAGE and Western Blotting

Figure 11 demonstrates BBB model integrity by detection of the TJ protein ZO1 in the different BBB models shown in Figure 1, Figure 2, Figure 3, Figure 4, Figure 5 and Figure 6 (Section 3.1). Figure 12 shows the relative protein expression of ZO1 compared to endothelial cells grown on no substratum.

### 3.5. Sizing and PDI

Table 1 provides information on PDI and size of PLGA NP in comparison to Fitc-dextran PLGA NP.

### 3.6. Encapsulation Efficiency

Table 2 provides information on the encapsulation efficiency (EE) of Fitc-dextran encapsulated within the PLGA NP.

### 3.7. Release

A release study was performed to determine cumulative release (%) of Fitc-dextran from the PLGA NP, as shown in Figure 13 where 1.5 mg of Fitc-dextran NP was dispersed in buffers of different pHs (DMGA pH 4.5, DPBS pH 7.1) to determine the influence of pH on the release of Fitc-dextran from PLGA NP over 24 h. 

### 3.8. Delivery across BBB Model

Figure 14 demonstrates the ability of DAS-Fitc-dextran PLGA NP as a vehicle to successfully transport and release a large hydrophilic compound (Fitc-dextran) (70,000 Da) across the optimal BBB model compared to Fitc-dextran PLGA NP. The NP were dispersed in SFM and added to the apical layer of the BBB model. At the 24 h timepoint, samples were removed from the basolateral layer of the BBB model inserts and analysed by fluorimetry (exc. 485 nm, em. 520 nm). Fitc-dextran PLGA NP and DAS-Fitc-dextran PLGA NP were tested on the optimal BBB model (D EP + A F + G Mg^2+^). Figure 14 also demonstrates that transport of the DAS-labelled NP across the BBB model can be blocked by the addition of α-7 nicotinic acetylcholine receptor antibody, mecamylamine and hexamethonium.

## 4. Discussion

### 4.1. In Vitro BBB Construction

When developing novel therapeutics, delivery and efficacy of the drugs must be determined before going forward. For assessment of the efficacy of drug-encapsulated NP, such as a DDS, we developed a cost-effective, easily manipulated, human in vitro BBB model that closely mimics the in vivo BBB architecture. In vitro models are an indispensable aid for drug permeability and toxicity studies, ensuring only lead compounds are brought forward to preclinical studies, reducing the cost and time frame of in vivo studies [27]. When developing an in vitro BBB model, cell selection and placement, choice of substratum and inclusion of shear stress must be considered to ensure the model achieves suitable integrity, which is determined by TEER values in excess of 150 Ω/cm^2^ and TJ protein expression [38]. Transepithelial electrical resistance (TEER) of a cell monolayer is a widely accepted quantitative measure of in vitro barrier integrity. TEER measurement can be performed on real-time assays with no detrimental effects on cell viability. A high TEER value suggests that paracellular transport of molecules is severely limited, thereby reducing the non-specific permeability of the in vitro model [39]. Models producing TEER values below 150 Ω/cm^2^ are deemed to have deceased integrity and will be prone to paracellular transport [39,40,41,42,43]. The addition of fluid shear stress to in vitro models allows the model to mimic the in vivo BBB more accurately by promoting cell elongation, which encourages TJ protein expression, enhancing the in vitro BBB function [44,45,46]. In vivo, shear stress has been shown to vary between 5 and 23 dny/cm^2^. In vitro models that apply shear stress above 5 dny/cm^2^ may cause cell detachment, and the most effective shear stress applied to in vitro models has shown to be 1.5 dny/cm^2^. Therefore, we utilised 1.5 dny/cm^2^ shear stress for our in vitro model [44,47,48]. 

**Figure 11:**
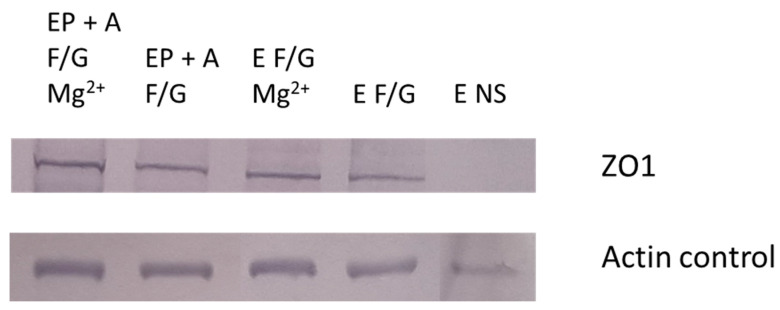
Image of Western blot membrane, detecting ZO1 TJ-protein (mw 195 kDA) in lysed cells from different in vitro BBB models. Endothelial cells (E), Pericytes (P), Astrocytes (A), Fibronectin + Gelatin (F + G), Fibronectin + Collagen (F + C), No substrata (NS) and Magnesium (Mg^2+^), Dynamic mode.

**Figure 12:**
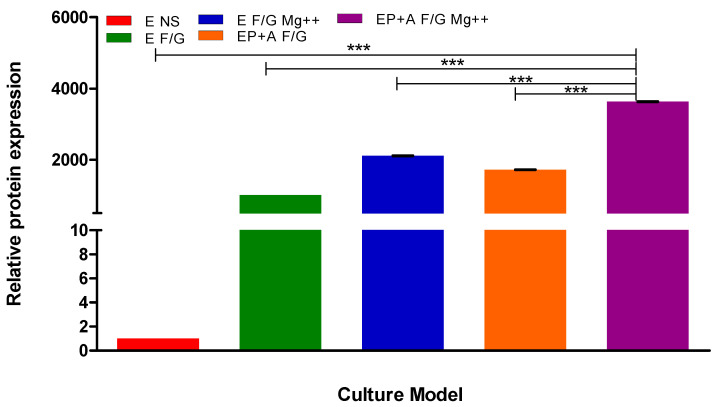
Quantitative analysis of relative protein expression of ZO1 in each BBB model. Endothelial cells (E), Pericytes (P), Astrocytes (A), Fibronectin + Gelatin (F + G), Fibronectin + Collagen (F + C), No substrata (NS) and Magnesium (Mg^2+^). *n* = 3, *** = *p* < 0.001; statistical analysis was performed using one-way ANOVA Bonferroni post hoc tests.

Immortalised cells were used for this model due to a reduced risk of contamination with other cell types (a problem when using primary and stem cells) and their cost-effectiveness. However, they have been deemed ‘leaky’ due to the low TEER values and low TJ protein expression obtained from monolayer in vitro BBB models [42,49]. Therefore, Figure 2, Figure 3, Figure 4, Figure 5, Figure 6, Figure 7, Figure 8, Figure 9, Figure 10, Figure 11 and Figure 12 describe the process of developing and optimising the ideal in vitro model to test our drug-encapsulated NP, by mimicking the in vivo BBB more accurately to increase TEER and TJ expression. Figure 2 shows how the addition of substratum compared to no substratum (N/S) influences TEER values. Substrata are used to mimic the basement membrane (BM) of the BBB. Substrata are forms of extracellular matrix (ECM) proteins which are found within the BM in vivo and are natural ligands for the BECs. They promote cell anchoring, give structural support, and promote signal transduction and are an important factor to consider when constructing an in vitro BBB model [50,51,52]. The optimal substrata chosen were a combination of fibronectin (F), an ECM protein found in vivo *and* gelatin (G), the denatured form of collagen, which has an exposed backbone allowing increased cell adhesion and increased BBB stability [50,51,52,53]. The addition of these substrata to the apical surface of the transwell inserts increased cell adhesion and therefore TEER. A study carried out by Maherally et al. (2018) explains the importance of ECM proteins and how certain ECM proteins stabilise TJ proteins and increase TJ expression, and this is in agreement with our results in Figure 2 [54]. The addition of shear stress on an endothelial cell monolayer did not increase TEER levels. Therefore, to mimic the in vivo BBB more accurately, we produced a tri-culture model utilising the substrata discussed previously (Figure 3 and Figure 4). The cells used and cell placement in tri-cultures replicate the NVU configuration and proximity to BECs which occur in vivo. Astrocytes and pericytes interact with the BECs and promote TJ protein expression and cellular transportation [32,35,55,56,57]. To increase the promotion of TJ proteins and, in turn, increase TEER, the cells were placed in close proximity to one another, along with the substrata and applied shear stress. In Figure 3 and Figure 4, substrata were pre-coated on the apical surface of the transwell insert, with IA and IP cell concentrations of 2 × 10^4^ cells/mL seeded to the basolateral surface of the transwell inserts.

From the results shown in Figure 3 and Figure 4, the dynamic (D) triculture model EP + A grown on fibronectin and gelatin substrata produced a higher TEER value but did not reach the gold standard of ≥150 Ω/cm^2^. The results show that the TEER is higher due to the application of shear stress, which is in agreement with Ferrell et al. (2019) and Elbakary et al. (2020). Their research has shown that shear stress on different cell types has increased protein expression, cell elongation and cell orientation, increasing TEER and barrier integrity [45,46]. Therefore, we kept the application of shear stress but altered the cell concentration of both the IA and IP (increased to 4 × 10^4^ cells/mL) and pre-coated both apical surface and the basolateral surfaces of the inserts to mimic the basement membrane more accurately (Figure 5). Figure 5 compares the TEER of an endothelial cell (E) monolayer BBB to that of the dynamic EP + A F + G model which produces a TEER value almost three times higher (approx. 200 Ω/cm^2^) than that of the monolayer model (approx. 70 Ω/cm^2^). Zhu et al. (2018) and Leon et al. (2021) have suggested that elevated magnesium levels in the culture medium can enhance BBB activity by significantly reducing the permeability of the barrier by regulating its function in vitro [58,59]. Figure 6 shows that the addition of 10 mM MgSO_4_ to the culture medium increases the TEER value of both the D EP + A F + G BBB model and on the endothelial cell monolayer. Because the addition of Mg^2+^ to the medium increased the TEER of the D EP + A F + G BBB model (230 Ω/cm^2^); it was decided that to produce the optimal in vitro BBB model, the addition of Mg^2+^ was required. 

Figure 7 illustrates the difference in TEER of each model, to show how the adjustments made to mimic the architecture and physiology of in vivo BBB structure produce an optimal model (D EP + A F + G Mg^2+^) to be utilised for the assessment of non-invasive drug delivery systems.

To test the permeability of the optimal BBB model, a large molecular weight compound was utilised, which would not permeate the BBB model via intercellular or paracellular routes, due to its hydrophilic nature. The compound used was Fitc-CM-dextran, which has a molecular weight of 70,000 Daltons. To test DAS’s ability to transport a large hydrophilic compound across the BBB, DAS was conjugated to Fitc-CM-dextran and tested alongside Fitc-CM-dextran on our optimal model with results shown in Figure 8 and Figure 9. DAS is an 18 amino acid ligand, which has an affinity for alpha 7 nicotinic acetylcholine receptors (α-7 nAChR) [37] found on endothelial cells of the BBB. It allows NP conjugated to the peptide to be transported transcellularly by RMT and is, therefore, an ideal mode to transport large hydrophilic molecules (such as antibodies) into the brain [7,22,23,24]. Figure 8 demonstrates that our optimal model has high integrity and low paracellular permeability as only 10% Fitc-CM-dextran permeated the BBB after 24 h, which shows that paracellular transportation is severally restricted. The Fitc-CM-dextran conjugated with DAS showed a higher percentage of compound to traverse the BBB (approximately 50%) after 24 h, indicating that the DAS is facilitating the transportation of Fitc-CM-dextran. Huey et al. (2019) developed the DAS ligand, and their study indicated that DAS, which was designed around a 5-mer sequence from RDP, had an affinity for the α-7 nAChR, and showed good stability in human serum and enhanced transport across the BBB via RMT in in vitro models compared to previous ligands [37]. This also agrees with results shown in Figure 9, which shows the statistically significant difference in transport efficacy which started to occur after one hour, with a significant difference of *p* < 0.05, and after 2.5 h, the significant difference increases to *p* < 0.001. Overall, these results from TEER measurement (Figure 7) and permeability assays, demonstrate that our optimal model has high integrity and low paracellular permeability, and that by conjugating the DAS ligand to a large molecule, it can aid transcellular transportation via RMT.

Immunocytochemistry was carried out to confirm the presence of IA and IP grown together on a glass coverslip, after the visualisation of cells with GFAP and αSMA antibodies, respectively [60,61,62,63] (Figure 10). DAPI-stained nuclei are visible in both cell types (Figure 10A,B).

In Figure 10, both IA and IP are visible, clearly demonstrating that cells can be co-grown in the same space; an important factor for the construction of our optimal model (where both IA and IP are grown on the underside of the transwell insert).

The increased TEER in our optimal model is due to the increased expression of TJ protein. Isolated proteins were analysed by SDS PAGE and Western blotting to show the expression of the ZO1 (Figure 11). ZO1 is a cytoplasmic protein, which is crucial for increasing BBB integrity as every TJ protein must interact with it to reduce the permeability of the BBB [3,35,64]. The top image demonstrates that ZO1 is expressed by I-HBMECs and that, depending on the model, different concentrations of ZO1 are expressed in the models. Figure 11 clearly demonstrates that the highest ZO1 expression levels are seen in our optimal model. The ZO1 bands for each model were quantified using Image J (Figure 12) to show the relative expression of ZO1 in the different models compared to endothelial cells grown as a monolayer. It can be clearly seen that over 3500 times the amount of ZO1 is produced by our optimal model compared to monolayer endothelial cells. Research carried out by Eigenmann et al. (2013), compared TEER and TJ-protein expression in four immortalised endothelial cell lines. They showed that TJ proteins had been expressed such as ZO1, but in varying degrees depending on the cell line. They also mentioned that the inclusion of pericytes and astrocytes did not increase TJ protein expression in immortalised endothelial cells [49]. However, when comparing their findings to our results in Figure 11 and Figure 12, we clearly demonstrate ZO1 levels increase with the presence of pericytes and astrocytes. This could be due to the presence of shear stress and substrata proteins that are found within the NVU, so our model more accurately resembles the in vivo NVU and, therefore, promotes TJ proteins expression due to cell anchoring and adhesion of pericytes and astrocytes in close proximity to BECs. Therefore, the results obtained in Figure 11 and Figure 12 support the TEER results, and it was concluded that the D EP + A F + G Mg^2+^ model was our optimal in vitro BBB model going forward.

### 4.2. Nanoparticle (NP) Development

NP were developed using poly (D, L-lactide-co-glycolide) (PLGA) and stabilised using 1.25% PVA solution. PLGA has been approved by the US FDA for sustained and controlled drug delivery systems as it is biodegradable, biocompatible and has low toxicity [65,66]. PLGA NP are not only ideal for encapsulating large hydrophilic molecules but the surface of PLGA NP can be chemically modified for the conjugation of ligands. Ligands on the surface of PLGA NP recognise and target the receptors found on the BECs and then traverse the BBB via RMT [67]. When developing PLGA NP for encapsulation of large molecules, the size of the PLGA NP should be in the range of 200–400 nm to have the ability to encapsulate the high mass of payloads. Chigumira et al. (2015) used PLGA NP to encapsulate pralidoxime and obtained size ranges between 300 and 400 nm [65]. Azizi et al. (2013) encapsulated bovine serum albumin (BSA) within PLGA NP of sizes 251.3 ± 8.5 nm [68]. BSA is a large globular protein with a molecular weight of 66 kDa, which is a similar molecular weight to that of Fitc-dextran which we encapsulate within our PLGA NP [69]. Our preferred size is between 200 and 400 nm to ensure high encapsulation of large MW compounds and more effective release. The polydispersity index (PDI) determines the uniformity of the NP in solution, with NP PDI ideally being close to zero as this indicates reduced size distribution and reduces particle aggregation [70,71].

Table 1 demonstrates the size and PDI of both blank PLGA NP and Fitc-dextran-encapsulated PLGA NP. Blank PLGA NP have a size of 268.5 nm (±36.63) with a PDI of 0.22 (±0.052), and the Fitc-dextran PLGA NP have a size of 347.7 nm (±62.48) with a PDI of 0.35 (±0.153), which is similar to PLGA NP sizes obtained by Chigumira et al. (2015). Fitc-dextran PLGA NP have a larger size than blank PLGA NP due to the encapsulation of Fitc-dextran. The PDI is slightly higher for Fitc-dextran PLGA NP, but a PDI below 0.5 is preferred, and we have obtained that for both NP. The size of both NP shown in Table 1 is within the 200–400 nm range with acceptable PDI. Table 2 shows the encapsulation efficiency (EE%) of Fitc-dextran within the PLGA NP was between 60 and 65%. This result is more stable than that of Chigumira et al. (2015), whose encapsulation range varied between 28 and 70% EE [65]. Fornaguera et al. (2015) had an EE% of over 90% of loperamide within PLGA NP. This molecule has a molecular weight of 477 Da and therefore the size of the payload may have affected EE% [72]. Huey et al. (2019) used PLGA NP to encapsulate Fitc-dextran and obtained similar sizes and encapsulation efficiency (286.5 ± 11.3 nm, EE 77%); therefore, from the results gained in this paper, it was determined that the next step was to carry out a release in different pH buffers to see if pH affected the NP release [37].

**Figure 13:**
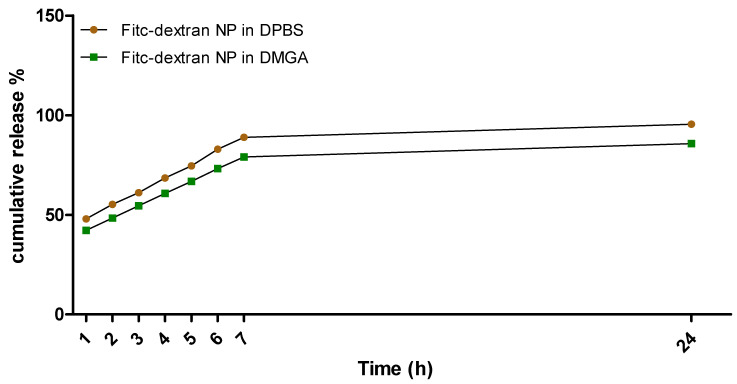
Cumulative release (%) of Fitc-dextran PLGA NP in DPBS and DMGA buffers over 24 h. *n* = 3 ± SD (exc. 485 nm, em. 520 nm). Statistical analysis was performed using two-way ANOVA Bonferroni post hoc tests, *p* < 0.001 comparing both buffers at each time point (error bars too small to be visualised on graph).

Figure 13 shows Fitc-dextran release from PLGA NP. At 1 h, Fitc-dextran released between 45 and 50% in both the DPBS buffer and DMGA buffer, with a gradual release up to 24 h. At 24 h, approximately 95% of Fitc-dextran has been released from the PLGA NP in DPBS and approximately 85% of Fitc-dextran has been released from the PLGA NP, proving that almost all the payload has released at 24 h irrespective of the pH of the release medium. This differs from the results of Patel et al. (2018), which show that pH does influence the release from PLGA NP [73]. They found that pH 5.5 buffer (after 48 h) had a higher release than pH 6.8 and pH 7.4 buffers; however, they only achieved a maximum release of 40% from PLGA NP at pH 5.5 [73]. When compared to our results in Figure 13, they also see an initial burst of release within the first couple of hours [73]. The variation between our release results and those of Patel et al. (2018) could be due to different PLGA NP formulation processes and payloads used. Therefore, it can be stated that the release results obtained within this paper demonstrate that PLGA NP are a suitable vehicle for sustained and controlled drug release of large hydrophilic compounds.

The NP-encapsulated Fitc-dextran payload will not be able to traverse the BBB unaided; therefore, the DAS ligand was attached to the NP to aid in the transportation via RMT. DAS was conjugated to the Fitc-dextran PLGA NP using a protocol developed by Huey et al. (2019). DAS was added in excess to the surface of the PLGA NP, which had been chemically modified by activating the free carboxyl groups on the surface of the PLGA NP using EDC/NHS in MES buffer [37]. As shown in Figure 8, DAS successfully aids in the transportation of large hydrophilic compounds across the BBB, which occurs via RMT utilising the α-7 nAChR found on the surface of BECs [37]. DAS Fitc-dextran PLGA NP and Fitc-dextran PLGA NP were tested on our optimal in vitro BBB model.

**Figure 14:**
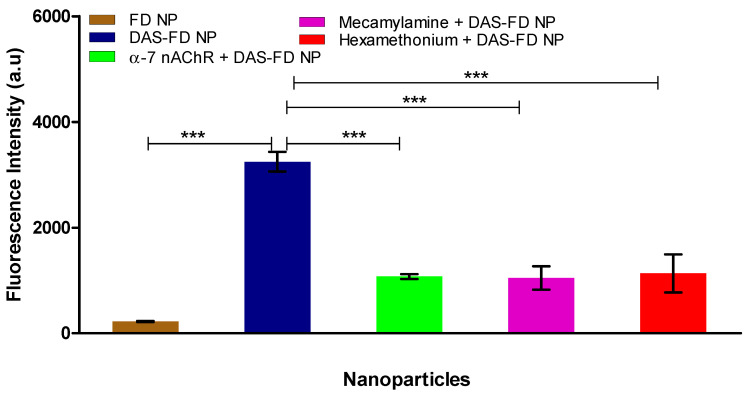
Fluorescence (a.u) intensity of the basolateral medium of the optimal BBB after delivery of Fitc-dextran NP and DAS-Fitc-dextran NP, after 24 h (exc. 485 nm, em. 520 nm), also in the presence of α-7 nicotinic acetylcholine receptor antibody, mecamylamine and hexamethonium. FITC NP = Fitc-dextran PLGA NP, DAS FITC NP = DAS conjugated to Fitc-dextran PLGA NP. *n* = 3 ± SD, *** = *p* < 0.001; statistical analysis was performed using one-way ANOVA Bonferroni post hoc tests.

Figure 14 shows the DAS Fitc-dextran PLGA NP were able to traverse the in vitro BBB model more successfully than the unconjugated Fitc-dextran PLGA NP, as the DAS Fitc-dextran PLGA NP had 14 times higher fluorescence intensity (a.u) (3200 a.u) than that of the unconjugated NP (225 a.u). We have also demonstrated that delivery of the DAS-labelled NP occurs via RMT, as this process can be blocked by the addition of an anti-α-7 nAChR antibody, mecamylamine (a nAChR non-competitive antagonist) and by hexamethonium (a nAChR competitive antagonist). Huey et al. (2019) showed the ability of DAS to bind to the α-7 nAChR and release a payload of Fitc-dextran within a monolayer of SH-SY5Y neuroblastoma cells [37]. Our results substantiate what Huey et al. (2019) visualised, confirming that DAS does have the ability to transport loaded PLGA NP across the BBB by RMT targeting the α-7 nAChR.

## 5. Conclusions

In conclusion, the optimal in vitro BBB model D EP + A F + G Mg^2+^, which utilises immortalised human cells, is an ideal, cheap, reproducible and effective method to test novel drug delivery systems for the CNS, such as ligand-targeted nanoparticulate systems. It has also been established that PLGA NP, which are easily developed, can successfully encapsulate and release large hydrophilic compounds. Finally, DAS-conjugated PLGA NP have been determined to be a suitable vehicle to successfully transport and release a large hydrophilic compound across the BBB by RMT. This targeted drug delivery system is a non-invasive effective method of transporting large hydrophilic payloads, such as therapeutic antibodies, to the CNS for the treatment of neurological disorders.

## Figures and Tables

**Table 1:** Sizing and PDI results gained from blank and Fitc-dextran encapsulated NP. *n* = 3 ± SD.

PLGA NP	Size (nm) ± SD	PDI ± SD
Blank PLGA NP	268.5 ± 36.63	0.22 ± 0.052
Fitc-dextran-PLGA NP	347.7 ± 62.48	0.35 ± 0.153
DAS-Fitc-dextran-PLGA-NP	386.50 ± 11.30	0.27 ± 0.08

**Table 2:** Encapsulation efficiency (EE) of Fitc-dextran in PLGA NP and conjugation efficiency (CE) of DAS to Fitc-dextran-PLGA NP.

Fitc-Dextran PLGA NP	EE%	CE%
Sample 1	64.49	62.50
Sample 2	61.87	64.06
Sample 3	64.94	56.80

## Data Availability

The data presented in this study are available on request from the corresponding author. The data are not publicly available due to privacy restrictions.
